# EVOLVING AGE-FRIENDLY UNIVERSITIES: ENHANCING STUDENT SUPPORT SERVICES AND PROMOTING SKILL DEVELOPMENT

**DOI:** 10.1093/geroni/igac059.1407

**Published:** 2022-12-20

**Authors:** Allyson Graf, Katherina Terhune, Heidi Ewen

## Abstract

Students in higher education face numerous challenges, many of which have been exacerbated by the pandemic. Globally, universities have capitalized on age-friendly programs and practices to respond to rapid changes in age demographics, build more age-inclusive and intergenerational programming, and create new forms of support campus-wide. This symposium features campus leaders representing universities that have drawn on the Age-Friendly University (AFU) principles to generate new and creative forms of support and skill-building for students of all ages. These initiatives represent the diverse ways that AFU-based programs and practices can be utilized to respond to opportunities and challenges in higher education. Graf et al. will discuss how age and age-related bias impact adult learners’ experiences in the classroom, and ways to inform training programs for students, faculty, and staff. Felsted and Eaton will describe a GSA/AARP-funded grant initiative aimed at increasing access, inclusion, and support for older adult learning at the university, resulting in a partnership with Emeritus faculty to pursue the AFU designation. Hancock and Kutcher will describe how service-learning and intergenerational learning opportunities provide a meaningful space for students to develop realistic notions of their own aging experiences. Newsham et al. will discuss a virtual intergenerational mentoring project designed to improve social connectedness and well-being, to improve expectations regarding aging, and to decrease ageism. Terhune et al. will discuss campus-wide data used to assess the prevalence and impact of unpaid caregiving on students and will explore community and campus connections to better support students in caregiving roles.

